# SAR Image Registration: The Combination of Nonlinear Diffusion Filtering, Hessian Features and Edge Points

**DOI:** 10.3390/s24144568

**Published:** 2024-07-14

**Authors:** Guili Tang, Zhonghao Wei, Long Zhuang

**Affiliations:** Nanjing Research Institute of Electronics Technology, Nanjing 210039, China; weizhh@163.com (Z.W.); zhuanglong14@163.com (L.Z.)

**Keywords:** synthetic aperture radar (SAR), image registration, infinite symmetric exponential filter (ISEF), nonlinear diffusion filtering, Hessian features, edge points

## Abstract

Synthetic aperture radar (SAR) image registration is an important process in many applications, such as image stitching and remote sensing surveillance. The registration accuracy is commonly affected by the presence of speckle noise in SAR images. When speckle noise is intense, the number of image features acquired by single-feature-based methods is insufficient. An SAR image registration method that combines nonlinear diffusion filtering, Hessian features and edge points is proposed in this paper to reduce speckle noise and obtain more image features. The proposed method uses the infinite symmetric exponential filter (ISEF) for image pre-processing and nonlinear diffusion filtering for scale-space construction. These measures can remove speckle noise from SAR images while preserving image edges. Hessian features and edge points are also employed as image features to optimize the utilization of feature information. Experiments with different noise levels, geometric transformations and image scenes demonstrate that the proposed method effectively improves the accuracy of SAR image registration compared with the SIFT-OCT, SAR-SIFT, Harris-SIFT, NF-Hessian and KAZE-SAR algorithms.

## 1. Introduction

Synthetic aperture radar (SAR) is an active microwave sensor for obtaining high-resolution images [1]. It has a high penetration ability and can work both day and night regardless of the weather conditions [2]. Due to these characteristics, SAR images are employed in many applications, such as change detection, image fusion and 3D reconstruction [3,4,5]. SAR image registration is an essential process of the mentioned applications. It aims to geometrically register SAR images acquired at different times from different perspectives or sensors [6,7,8].

Generally, SAR image registration methods can be categorized as intensity-based methods and feature-based methods [9]. Intensity-based methods usually measure regional similarity with mutual information (MI) [10] or normalized cross-correlation (NCC) [11]. These methods are predominantly reliant on image grayscale, rendering them highly susceptible to noise and geometric deformation. Feature-based methods register images by identifying the correspondence of reliable feature pairs [12], like points, lines, edges and so forth. These methods are robust to grayscale changes, resistant to geometric deformation and used widely in SAR image registration.

Feature-based methods such as scale-invariant feature transform (SIFT) [13] and speeded up robust features (SURF) [14] perform well at matching optical images. However, these methods do not perform as well as we would like for SAR image registration because the substantial multiplicative noise in SAR images introduces significant interference in feature detection. Most feature-based methods are designed based on the SIFT framework. Many improvement methods have been proposed to make the SIFT framework applicable to SAR images. Generally, these improvements come from two aspects: scale-space construction and feature detection.

In the aspect of scale-space construction, the SIFT-OCT [15] algorithm skips the first octave of image scale space used in SIFT for feature detection, which reduces the detection time and improves the correct matching ratio. The BF-SIFT algorithm [16] uses bilateral filters (BFs) to build image scale space and implements the dual-matching strategy to match features. Fan et al. [17] constructed the multi-scale space using nonlinear diffusion filters and utilized phase congruency information to remove erroneous feature points. Wang et al. [18] proposed an adapted anisotropic Gaussian SIFT (AAG-SIFT) algorithm to obtain stable and precise matching features for SAR image registration.

In the aspect of feature detection, the SAR-SIFT [19] algorithm employs the ratio of exponentially weighted average (ROEWA) for image gradient computation and multi-scale Harris detectors for feature detection. The polar scale-invariant feature transform (PSIFT) descriptor [20] is proposed to describe the features precisely. Chang et al. [21] proposed a Fourier histogram of oriented ratio gradient to determine the main orientation, which mitigated the impact of speckle noise on the principal orientation assignment. Peng et al. [22] combined SIFT features with Harris corners to register images for 3D reconstruction and obtained much finer 3D information. Ye et al. [23] proposed a point feature detector that combines blob features and Harris corners to improve the registration accuracy of remote sensing images. The KAZE-SAR [24] algorithm modifies the SURF descriptor in the KAZE [25] algorithm to register SAR images more accurately.

Considering scale-space construction and feature detection, this paper proposes a dual-feature SAR image registration algorithm which combines nonlinear diffusion filtering, Hessian features and edge points. This algorithm has made the following improvements to the SIFT framework. First, the infinite symmetric exponential filter (ISEF) [26] is applied for image pre-processing, and nonlinear diffusion filtering is employed for scale-space construction. These measures can remove speckle noise while preserving image edges. Meanwhile, to further suppress the correlated noise, the ROEWA operator is employed for gradient computation. Second, the Hessian features [27] and edge points are jointly used as image key points, which optimizes the utilization of feature information in SAR images. Therefore, the objective of the proposed method is to tackle the problem of severe speckle noise and limited image features in SAR image registration. Experiments with different noise levels, geometric transformations and image scenes demonstrate that the proposed method effectively improves the registration accuracy of SAR images compared with the SIFT-OCT, SAR-SIFT, Harris-SIFT, NF-Hessian and KAZE-SAR algorithms. The contributions of this paper are as follows.

(1) A multi-scale space construction strategy that integrates nonlinear diffusion filtering into the SIFT scale space is designed. It achieves satisfactory performance in edge preservation and speckle noise removal.
(2) A novel feature detection method combining Hessian features and edge points is proposed to obtain more matching feature points. The GLOH descriptor is employed for feature description to accurately describe the features.
(3) ISEF is applied for image pre-processing to preserve the edge details on the initial SAR image. The ROEWA operator is used for gradient computation to suppress multiplicative noise.

The subsequent sections are structured as follows: Section 2 clarifies the essential principles and theories related to the proposed method. Section 3 provides a detailed description of the experimental setup and displays the registration results. Finally, Section 4 restates the process of the proposed method, summarizes the experiment results and discusses the implications and limitations.

## 2. Principles of the Proposed Algorithm

**Figure 1:**
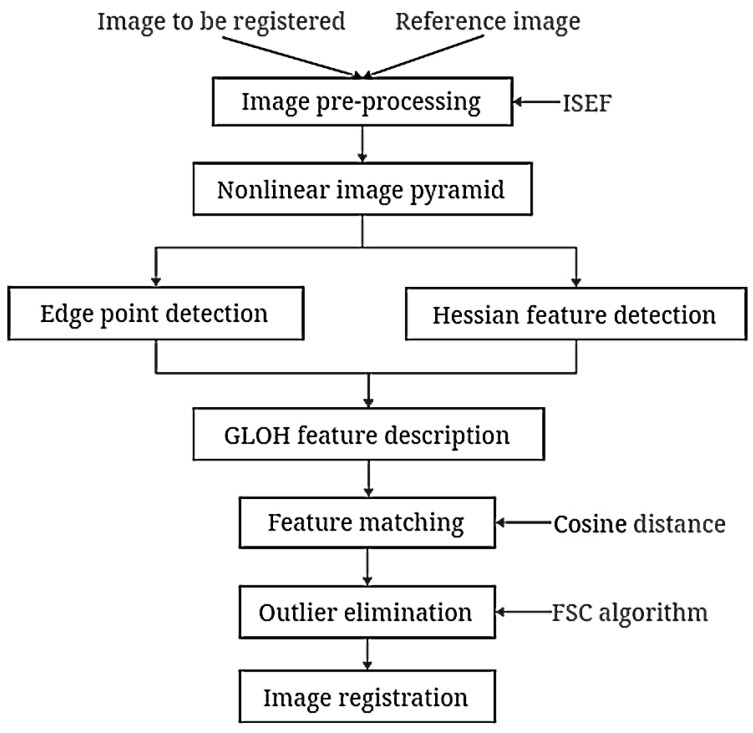
The flowchart of the proposed algorithm.

This section elaborates on the principles of the proposed algorithm. This algorithm encompasses four crucial operations: image pre-processing based on the infinite symmetric exponential filter (ISEF) [26], scale-space construction based on nonlinear diffusion filtering, feature detection combining Hessian features and edge points, and feature description and matching. The flowchart of the proposed algorithm is depicted in Figure 1.

### 2.1. Image Pre-Processing Based on ISEF

Image pre-processing aims to suppress speckle noise and enhance image features. The simplest method for image pre-processing is applying a Gaussian filter to the initial images. However, this approach results in the blurring of image edges and the loss of structural information. To preserve the image edges and details better, ISEF is applied for image pre-processing.

The convolution kernel of ISEF is formulated as

(1) $$G\left(x,y\right)=e^{-\frac{\left|x\right|+\left|y\right|}{\sigma }},$$

where σ is the standard deviation (STD). The peak value of the ISEF kernel $G\left(x,y\right)$ is not equal to 1. To limit the grayscale range within 0~255, the peak value of this filter kernel should be normalized when used.

**Figure 2:**
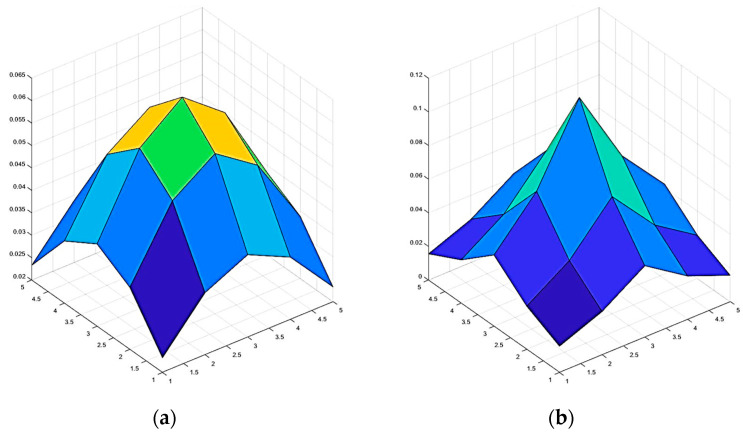
The shape of the Gaussian filter and ISEF. (**a**) Gaussian filter with σ = 2. (**b**) ISEF with σ = 2.

**Figure 3:**
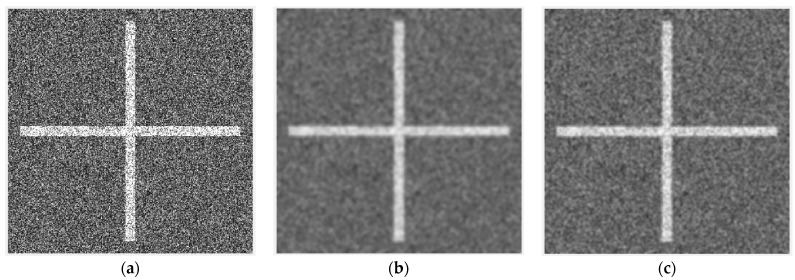
Filtering results of Gaussian filter and ISEF. (**a**) Original image. (**b**) Image filtered by Gaussian filter. (**c**) Image filtered by ISEF.

Figure 2 depicts the shape of a Gaussian filter and ISEF. The STD of both filters is 2. Compared with the Gaussian filter, ISEF assigns lower weights to the surrounding pixels to mitigate the blurring of image edges. Figure 3 exhibits the filtering results of both filters, which demonstrate that ISEF is more effective in preserving image edges.

### 2.2. Scale-Space Construction Based on Nonlinear Diffusion Filtering

The scale space of the SIFT algorithm is constructed by blurring the images with Gaussian filters at different scales. However, Gaussian filtering suffers from the blurring of edges and the loss of details. Compared with a Gaussian filter, a nonlinear diffusion filter [28] is more advantageous in removing speckle noise and preserving edges. Thus, we replaced the Gaussian filters with nonlinear diffusion filters to generate image scale space.

The nonlinear diffusion equation is formulated as

(2) $$\frac{\partial L}{\partial t}=div[C\left(x,y,t\right)\nabla L],$$

where L represents the image to be processed, t is a scale-dependent value, div denotes the divergence operator, C is the diffusion coefficient and ∇ is the gradient operator.

The value of t is defined as

(3) $$t_{n}=\frac{1}{2}(\sigma_{n}^{2}-\sigma_{n-1}^{2}),$$

where $\sigma_{n}$ and $\sigma_{n-1}$ represent the variance of the current and the previous image layer, respectively.

The diffusion coefficient C is highly correlated with image gradient ∇L. Two classic diffusion coefficient functions [28] are defined as follows:

(4) $$C_{1}=\exp(-\frac{{\left|\nabla L\right|}^{2}}{k^{2}}),$$

(5) $$C_{2}=\frac{1}{1+\frac{{\left|\nabla L\right|}^{2}}{k^{2}}},$$

where k is a coefficient related to the contrast ratio. The value of k is generally in the 70% quantile of the gradient histogram.

The function (5) is chosen for diffusion filtering. The image gradient ∇L can be obtained by applying a first-order difference operator to the image in question. However, this method tends to generate false edges in high-reflectivity regions due to the multiplicative noise present in SAR images. Consequently, the ROEWA operator, which is more suitable for SAR images, is employed to compute the image gradient ∇L.

There is no analytic solution for the nonlinear diffusion Equation (2). Hence, numerical methods are required to find an approximate solution. The semi-implicit scheme [29] is employed to discretize the nonlinear diffusion equation. The discretized equation is

(6) $$L^{n+1}=[I-\tau \sum_{l}^{m}A_{l}(L^{n}){]}^{-1}L^{n},$$

where $L^{n}$ and $L^{n+1}$ represent the n-th and the (n+1)-th filtered images, I is the unit matrix, τ denotes the time step of diffusion, m represents the image dimension, and $A_{l}$ is the matrix of diffusion coefficients in the l-th dimension.

When the matrix $A_{l}$ has a considerable size, the computation of Equation (6) will become exceedingly complex. The additive operator splitting (AOS) [29] algorithm can improve computational efficiency. By applying this algorithm, Equation (6) is rewritten as the following three formulas:

(7) $$2[I-2\tau A_{1}\left(L^{n}\right)]L_{1}^{n+1}=L^{n},$$

(8) $$2[I-2\tau A_{2}\left(L^{n}\right)]L_{2}^{n+1}=L^{n},$$

(9) $$L^{n+1}=L_{1}^{n+1}+L_{2}^{n+1},$$

where $A_{1}\left(L^{n}\right)$ and $A_{2}\left(L^{n}\right)$ denote the matrices of diffusion coefficients in rows and columns, and $L_{1}^{n+1}$ and $L_{2}^{n+1}$ represent the images after row diffusion and column diffusion.

Except for nonlinear diffusion filtering, the other parameters employed in the proposed method for scale-space construction are the same as the SIFT algorithm. It is essential to mention that the proposed method is not needed to compute Gaussian difference images.

**Figure 4:**
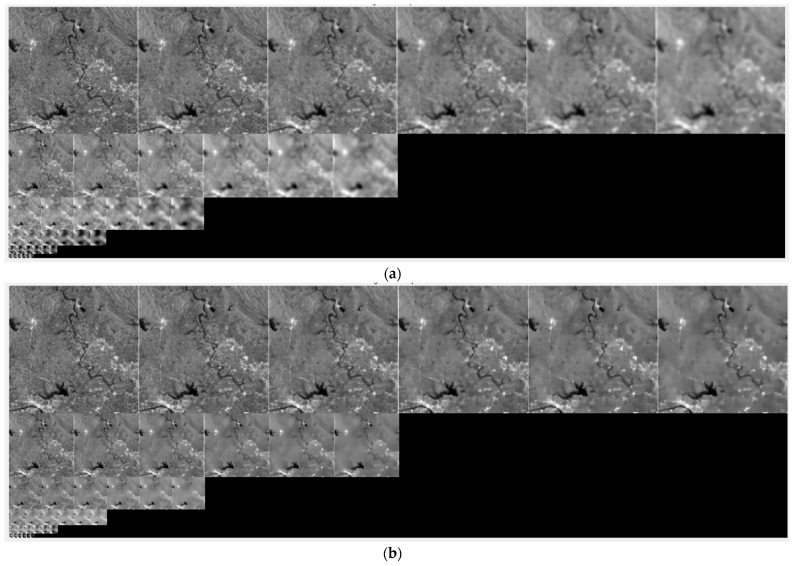
Scale spaces of SAR image. (**a**) Scale space constructed by Gaussian filtering. (**b**) Scale space constructed by nonlinear diffusion filtering.

Figure 4 presents a comparison of scale spaces constructed by Gaussian filtering and nonlinear diffusion filtering. As the number of filter iterations increases, the more speckle noise is removed by nonlinear diffusion filtering, the sharper the image edges become. Meanwhile, Gaussian filtering makes the image increasingly blurry. The scale-space results demonstrate that nonlinear diffusion filtering is more effective in preserving image edges and details.

**Figure 5:**
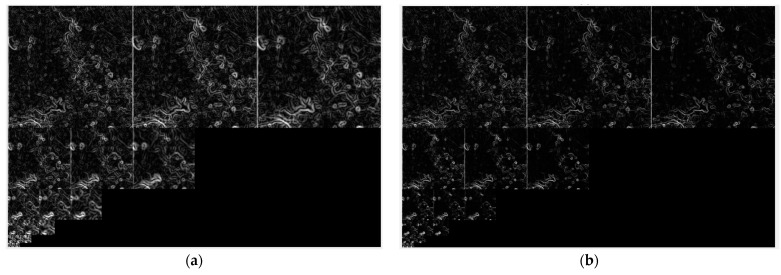
Gradient images acquired from the scale spaces above. (**a**) Gradient images generated by the SIFT algorithm. (**b**) Gradient images generated by the proposed algorithm.

Figure 5 shows the gradient images of the middle three layers of Gaussian and nonlinear scale spaces. The gradient images generated by the proposed algorithm are significantly more distinct than those produced by the SIFT algorithm.

### 2.3. Feature Detection Combining Hessian Features and Edge Points

The point features commonly extracted in nonlinear scale space are Hessian [30] and Harris [31] features. Harris features are generally located on image edges. Thus, the proposed method employs Hessian features and edge points as image feature points. The following subsections describe the detection of Hessian features and edge points.

#### 2.3.1. Hessian Feature Detection

A Hessian matrix is an n×n square matrix composed of the second-order partial derivatives of a function with n variables. Since images are two-dimensional, the Hessian matrix used for image processing is

(10) $$H=\left[\begin{array}{cc} \frac{\partial^{2}L}{\partial x^{2}} & \frac{\partial^{2}L}{\partial x\partial y}\\ \frac{\partial^{2}L}{\partial y\partial x} & \frac{\partial^{2}L}{\partial y^{2}} \end{array}\right]=\left[\begin{array}{cc} L_{xx} & L_{xy}\\ L_{yx} & L_{yy} \end{array}\right].$$

The determinant of the Hessian matrix reflects the local curvature in the vicinity of pixel point (x,y). The larger the determinant, the greater the curvature; the smaller the determinant, the lesser the curvature.

The Hessian response function is defined as

(11) $$F_{Hessian}=\sigma^{2}\left|H\right|=\sigma^{2}(L_{xx}L_{yy}-L_{xy}L_{yx}),$$

where $\sigma^{2}$ is the variance of the current image L. The reason for multiplying $\sigma^{2}$ here is to normalize the scale.

**Figure 6:**
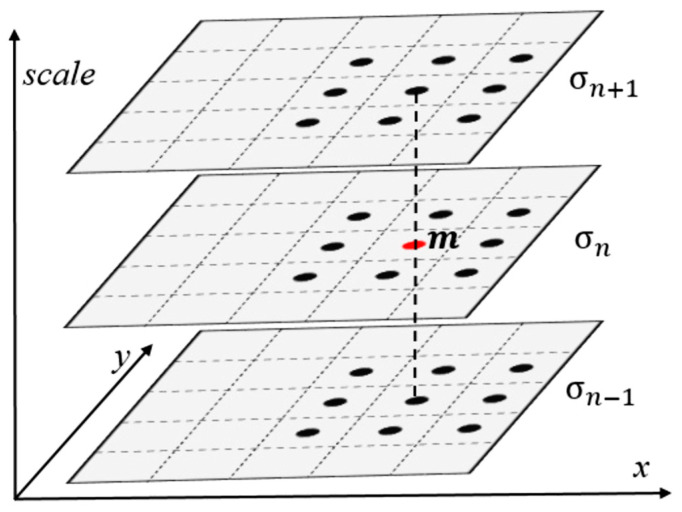
Diagram of Hessian feature detection [23].

A schematic of Hessian feature detection is depicted in Figure 6. The pixel m with Hessian response value $F_{Hessian}$ larger than the surrounding 26 points is defined as a Hessian feature point.

#### 2.3.2. Edge Point Detection

The image edges generally refer to the boundaries or transition areas between different regions with significant changes in gray value. It is usually described through the image gradient. The proposed method employs the ROEWA operator to compute the image gradient. The specific process of gradient calculation is presented below:

(12) $$R_{x}=\frac{\log\left(M_{x+}\right)}{\log\left(M_{x-}\right)}\;\;\;\;R_{y}=\frac{\log\left(M_{y+}\right)}{\log\left(M_{y-}\right)},$$

(13) $$G=\sqrt{{R_{x}}^{2}+{R_{y}}^{2}},$$

(14) $$\theta =\arctan\left(\frac{R_{x}}{R_{y}}\right),$$

where $R_{x}$ and $R_{y}$ denote the gradients in the row and column directions; $M_{x+}$, $M_{x-}$, $M_{y+}$ and $M_{y-}$ are the means of the gray values of the four regions: left, right, up and down.

Following the preceding operations, the points with small gradient values are first removed from the gradient images. Then, morphological operations are employed to refine the edges and eliminate components with small areas. Finally, the remaining pixel points are the desired edge points.

### 2.4. Feature Description and Matching

To make the detected features more rotationally invariant, the gradient location-orientation histogram (GLOH) [32] is utilized for feature description. The cosine distance is adopted to measure the similarity of features. Two features with high similarity are regarded as matching features. Finally, the mismatched features are then eliminated by the fast sample consensus (FSC) [33] algorithm. The affine transformation model is employed to register the images.

## 3. Experiments on SAR Image Registration

This section presents the results of three sets of SAR image registration experiments conducted to validate the performance of the proposed method. These experiments are SAR image registration under different noise levels, SAR image registration under different geometric transformations, and SAR image registration under different scenes. Further details for these experiments, such as data sets, evaluation indicators, comparison experiments and parameter settings, are provided below.

### 3.1. Data Sets and Evaluation Indicators

**Figure 7:**
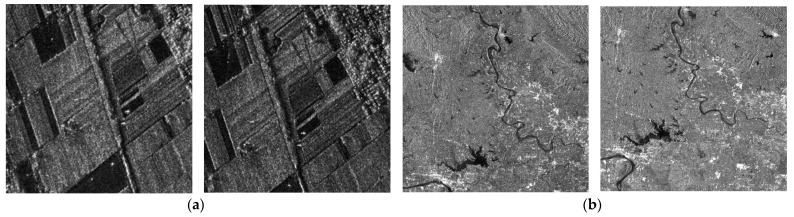

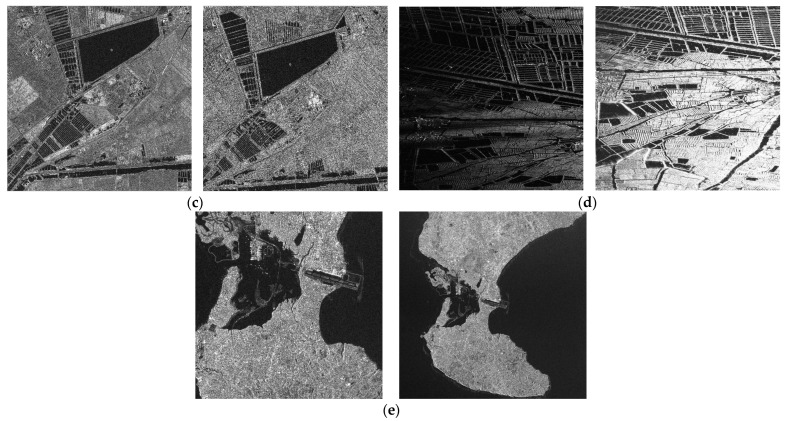
SAR images used in experiments. (**a**) Images with substantial corner points [24]. (**b**) Images with almost no corner points [24]. (**c**) Images with severe speckle noise [34]. (**d**) Images with different illuminations [35]. (**e**) Images with different scales [36].

The five pairs of SAR images in Figure 7 were used for the aforementioned experiments. Figure 7a,b were sourced from the link https://github.com/Pourfardm/SAR-image-registration-KAZE [24] accessed on 21 March 2024. Their satellite information is not provided in reference [24]. Figure 7a, 401 × 401 pixels, shows agricultural lands with substantial corner points. Figure 7b, 397 × 390 pixels, shows fluvial areas with few corner features. Figure 7c, 666 × 670 pixels, was acquired by Radarsat-2 in June 2009 and June 2008 [34], representing suburban districts with serious speckle noise. Figure 7d, 800 × 798 pixels, was obtained from two different SARs, a satellite and an unmanned aerial vehicle (UAV) [35]. It was captured in Nantong, Jiangsu Province, China, in 2013. Figure 7e, 512 × 512 pixels, was captured by the ESA Sentinel-1B satellite and depicts Ngurah Rai International Airport in Bali, Indonesia [36].

In this paper, the evaluation indicators for SAR image registration are correct matching number (CMN) [37], root mean square error (RMSE) [38], standard deviation (STD) [23] and computation time. These indicators are described in detail as follows:

(1) CMN represents the number of correct matching features. It is obtained by eliminating outliers from the initial matches with the FSC algorithm. The larger the value of CMN, the easier it is to obtain highly accurate registration results.
(2) RMSE and STD are computed by 20 pairs of checkpoints manually selected from both the reference image and the image to be registered. The specific calculation formulas for RMSE and STD are

(15) $$RMSE=\sqrt{\frac{1}{m}\sum_{i=1}^{m}\left[{\left(x_{i}-x_{i}^{\prime }\right)}^{2}+{\left(y_{i}-y_{i}^{\prime }\right)}^{2}\right]},$$

(16) $$STD=\sqrt{\frac{1}{m}\sum_{i=1}^{m}{{(error}_{i}-E[error])}^{2}},$$

(17) $${error}_{i}=\sqrt{{\left(x_{i}-x_{i}^{\prime }\right)}^{2}+{\left(y_{i}-y_{i}^{\prime }\right)}^{2}},$$

where m is the number of checkpoints, $\left(x_{i},y_{i}\right)$ is the coordinate of the *i*-th checkpoint on the reference image, and ${(x}_{i}^{\prime },y_{i}^{\prime })$ is the transformed coordinate of the *i*-th checkpoint on the image to be registered. RMSE and STD are essential parameters for registration accuracy measurement. The smaller their value, the higher the registration accuracy.

(3) Computation time is the duration from image pre-processing to the completion of image registration. The shorter the time, the more efficient the calculation.

### 3.2. Comparison Experiments and Parameter Settings

We selected the SIFT-OCT [15], SAR-SIFT [19], Harris-SIFT [22], NF-Hessian and KAZE-SAR [24] algorithms as comparison experiments. SIFT-OCT and SAR-SIFT were selected because they have been widely recognized and frequently cited in image registration studies. The Harris-SIFT algorithm, combining Harris and SIFT features for feature detection, is a referable comparison method based on dual features. The NF-Hessian algorithm, combining nonlinear diffusion filtering and Hessian features, is designed based on the KAZE [25] algorithm. It replaces the SURF descriptor used in KAZE with the SIFT descriptor and constructs the scale space by image down-sampling. The process and parameter settings of NF-Hessian are identical to those of the proposed method, except that edge points are not utilized. The KAZE-SAR algorithm uses the KAZE detector and modified SURF descriptor to tackle the speckle noise in SAR images. The KAZE-SAR algorithm and the proposed method employ the same nonlinear diffusion filtering but differ in scale-space construction and feature description. KAZE-SAR does not down-sample the images during scale-space construction, while the proposed method does. KAZE-SAR uses the modified SURF descriptor for feature description, while the proposed method uses the GLOH descriptor.

The experiments presented in this paper were all implemented in MATLAB 2018b using a laptop equipped with Intel Core i7-8550U CPU, 8 GB RAM (Intel, Santa Clara, CA, USA), a 1 TB solid-state drive (SSD) and a 64-bit operating system.

Here is a detailed introduction of the experimental parameters. In image pre-processing, the kernel size of ISEF is 5 × 5 pixels, while the standard deviation σ of ISEF is 0.5. In scale-space construction, the number of central layers in the image groups is 3, and the initial image scale is 1.6. In nonlinear diffusion filtering, the number of bars in the gradient histogram used to calculate the contrast factor k is 500. The percentile required for the calculation is 0.7. The image boundary protection size for feature detection is 2 pixels. The minimum value of the Hessian function response is limited to 0.33, and the edge threshold is 0.45. The number of bars in the histogram of gradient directions is 36, and the histogram peak ratio is 0.8. The maximum RMSE allowed for feature matching is 3 pixels. The transform matrix of the SAR images results from 500 iterations of the affine transform model.

### 3.3. SAR Image Registration under Different Noise Levels

The SAR image pair shown in Figure 7a with substantial corner points and edge features was selected for this experiment. To generate SAR images with different noise levels, multiplicative noise with different variances was added to the right image of Figure 7a. Then, SAR-SIFT, Harris-SIFT, NF-Hessian, KAZE-SAR and the proposed method were applied to register these images with the left image of Figure 7a. The SIFT-OCT algorithm was not employed here because it failed in registering Figure 7a.

**Figure 8:**
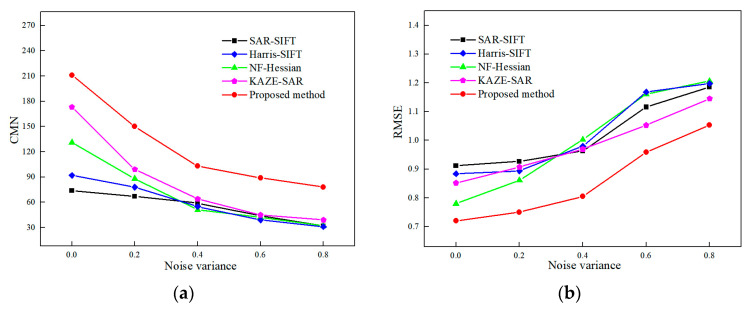
Registration performance under different noise levels. (**a**) CMN. (**b**) RMSE.

**Figure 9:**
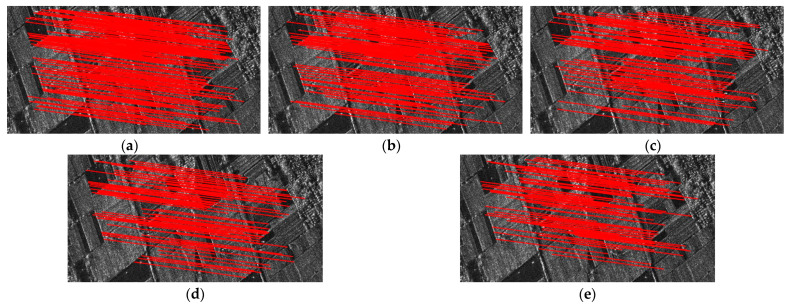
Matching feature points under different noise levels obtained by the proposed method. (**a**) No extra noise. (**b**) Added noise with variance of 0.2. (**c**) Added noise with variance of 0.4. (**d**) Added noise with variance of 0.6. (**e**) Added noise with variance of 0.8.

Figure 8 depicts the CMN and RMSE of SAR image registration under different noise levels. Figure 9 shows the matching feature points obtained by the proposed method. The performances of SAR-SIFT, Harris-SIFT, NF-Hessian, KAZE-SAR and the proposed method deteriorate as the noise variance increases. This phenomenon occurs because the multiplicative noise causes the generation of spurious feature points, which even mask the original image features.

The NF-Hessian algorithm and the proposed method employ nonlinear diffusion filters for noise suppression, which allows them to perform better than the SAR-SIFT and Harris-SIFT algorithms at lower noise levels. Although KAZE-SAR obtains relatively more feature points by nonlinear diffusion filtering and image-size preserving during scale-space construction, the small dimensionality of its feature descriptor causes larger errors in matching features. When the noise level rises, noise suppression by nonlinear diffusion filtering deteriorates. Since Harris features are more robust to noise than Hessian features, the performance of SAR-SIFT gradually approaches and exceeds that of the NF-Hessian algorithm. The SAR-SIFT algorithm achieves a lower RMSE than Harris-SIFT at high noise levels due to the noise resistance of the ROEWA operator. KAZE-SAR obtains higher CMN and lower RMSE at high noise levels compared with SAR-SIFT, Harris-SIFT and NF-Hessian. The proposed method combining Hessian features and edge points performs the best at different noise levels.

In conclusion, the proposed method outperforms the SAR-SIFT, Harris-SIFT, NF-Hessian and KAZE-SAR algorithms in terms of CMN and RMSE under different noise levels.

### 3.4. SAR Image Registration under Different Geometric Transformations

In this experiment, the images to be registered were obtained by rotating and scaling the right image of Figure 7a to different extents. SAR-SIFT, Harris-SIFT, NF-Hessian, KAZE-SAR and the proposed method were applied to register these transformed images with the left image of Figure 7a.

**Figure 10:**
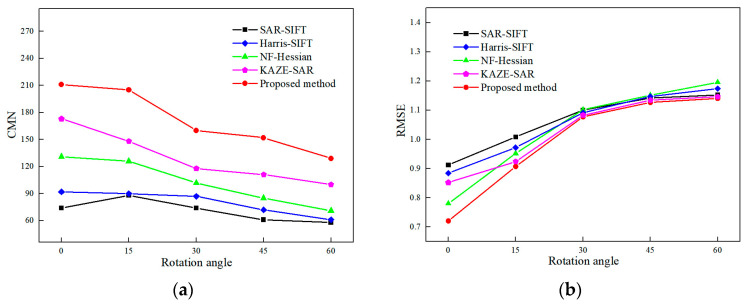
Registration performance under different rotation angles. (**a**) CMN. (**b**) RMSE.

**Figure 11:**
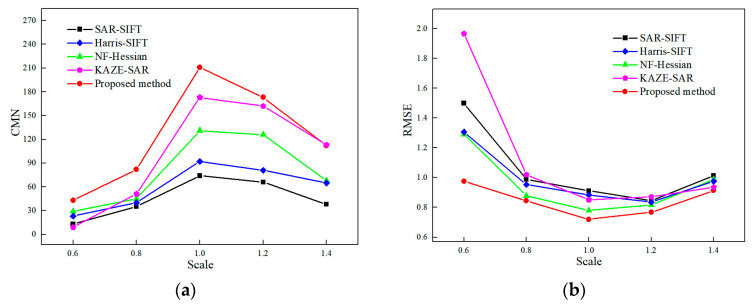
Registration performance under different scales. (**a**) CMN. (**b**) RMSE.

**Figure 12:**
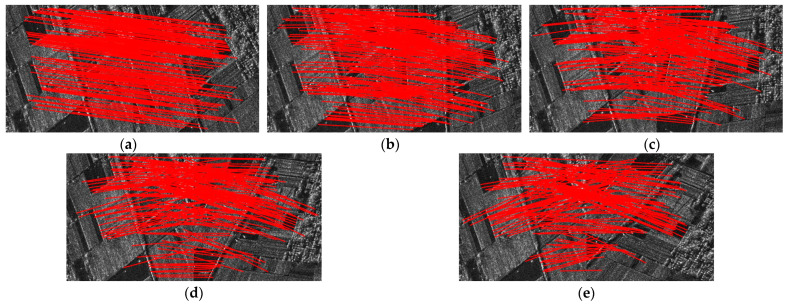
Matching feature points under different rotation angles obtained by the proposed method. (**a**) No rotation. (**b**) The rotation angle is 15 degrees. (**c**) The rotation angle is 30 degrees. (**d**) The rotation angle is 45 degrees. (**e**) The rotation angle is 60 degrees.

**Figure 13:**
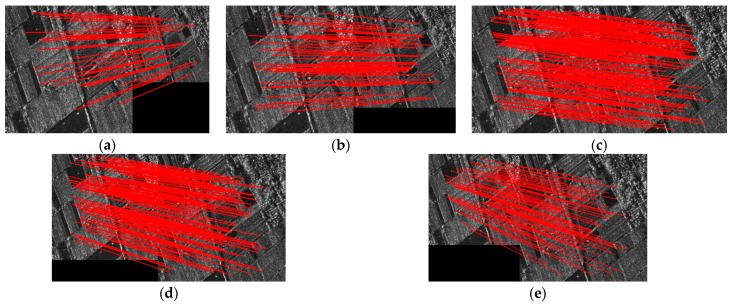
Matching feature points under different scales obtained by the proposed method. (**a**) Scale size of 0.6. (**b**) Scale size of 0.8. (**c**) Scale size of 1. (**d**) Scale size of 1.2. (**e**) Scale size of 1.4.

Figure 10 and Figure 11 display the experimental results of SAR image registration under different rotation angles and scales. These two figures demonstrate that the registration accuracy of all tested methods declines as the rotation angle increases or the scale difference widens. Figure 12 and Figure 13 show the matched feature points obtained by the proposed method under different rotation angles and scales.

Regarding rotation transformation, when the rotation angle is narrow, the effect of speckle noise on the registration results is more significant than that of rotation angles. NF-Hessian, KAZE-SAR and the proposed method, all of which employ nonlinear diffusion filtering, perform better than the SAR-SIFT and Harris-SIFT algorithms. As the rotation angle increases, the rotational invariance of the feature descriptor becomes increasingly crucial. SAR-SIFT and the proposed method use the GLOH operator for feature description, while Harris-SIFT and NF-Hessian still employ the SIFT descriptor. Thus, SAR-SIFT and the proposed algorithm obtain a lower RMSE than the other two methods at wide rotation angles. KAZE-SAR outperforms the SAR-SIFT, Harris-SIFT and NF-Hessian algorithms at wide rotation angles due to its improved SURF descriptor. The proposed method, combining nonlinear diffusion filtering and the GLOH operator, achieves optimal performance at different rotation angles.

Regarding scale transformation, scale-space construction is significant for image registration. The SAR-SIFT and KAZE-SAR algorithms do not down-sample the images during scale-space construction. This means the scale ranges of their feature detection spaces are narrow. Harris-SIFT, NF-Hessian and the proposed method construct image scale space by down-sampling the images, which makes them more robust to scale transformations. As a result, their registration accuracies at different scales are mostly higher than those of SAR-SIFT and KAZE-SAR. The Harris-SIFT algorithm combines Harris and SIFT features but still uses Gaussian filtering for scale-space construction. Its registration accuracy is between that of SAR-SIFT and NF-Hessian. In addition, the proposed method achieves larger CMN and lower RMSE than the Harris-SIFT, NF-Hessian and KAZE-SAR algorithms because it combines nonlinear diffusion filtering, Hessian features and edge points.

In conclusion, the proposed method obtains more matching feature points and achieves lower RMSE than the SAR-SIFT, Harris-SIFT, NF-Hessian and KAZE-SAR methods under different rotation or scale transformations.

### 3.5. SAR Image Registration under Different Scenes

In this experiment, SAR images (Figure 7) of different scenes were registered with the SIFT-OCT, SAR-SIFT, Harris-SIFT, NF-Hessian, KAZE-SAR algorithms and the proposed method. The objective is to assess the generalizability and superiority of the proposed method compared with the other methods.

**Figure 14:**
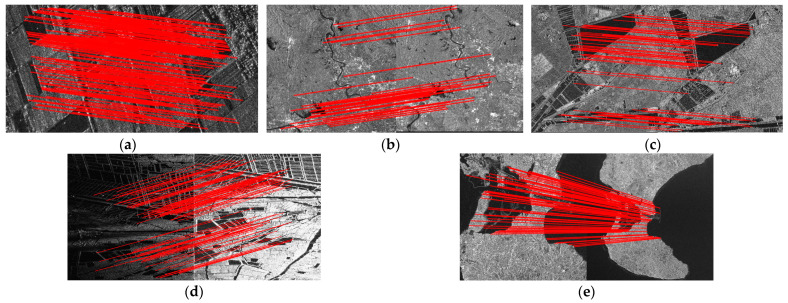
Matching feature points obtained by the proposed method. (**a**) Agricultural land. (**b**) Fluvial area. (**c**) Suburban district. (**d**) Different illuminations. (**e**) Different scales.

Figure 14 shows the matching feature points of these image pairs obtained by the proposed method. The proposed method obtains many matching feature points on the five different SAR images. The result demonstrates that the proposed method can register images from different sources, with different illuminations and at different scales.

**Figure 15:**
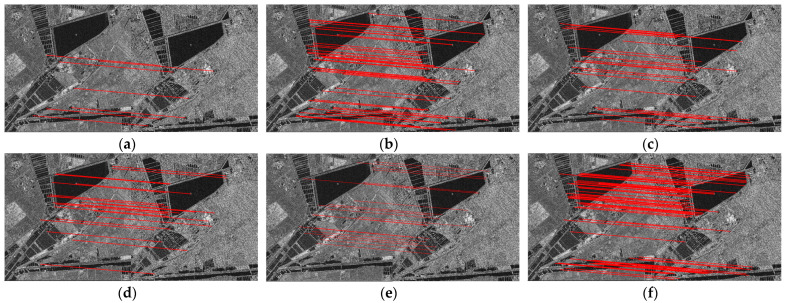
Matching feature points obtained by different methods. (**a**) SIFT-OCT. (**b**) SAR-SIFT. (**c**) Harris-SIFT. (**d**) NF-Hessian. (**e**) KAZE-SAR. (**f**) The proposed method.

Figure 15 displays the matching feature points on the images of a suburban district obtained by different methods. The proposed method obtains more matching feature points than the SIFT-OCT, SAR-SIFT, Harris-SIFT, NF-Hessian and KAZE-SAR algorithms on the images of the suburban district.

The results in Table 1 demonstrate that the proposed method is applicable to SAR images with fewer features, severe noise, different illuminations and different scales. The SIFT-OCT algorithm fails to register the images of agricultural lands and fluvial areas. The SAR-SIFT algorithm performs poorly when registering the images with few corner points. The NF-Hessian and KAZE-SAR algorithms fail to register the images of Bali’s airport, which exhibit large differences in scale. The CMNs of KAZE-SAR are higher than the proposed method in Figure 7b,d, but the RMSEs are higher than the proposed method. It indicates that the CMNs obtained by KAZE-SAR have lower credibility because its feature descriptors are not precise enough. The Harris-SIFT algorithm performs moderately in these five scenes. Compared with the SIFT-OCT, SAR-SIFT, Harris-SIFT, NF-Hessian and KAZE-SAR algorithms, the proposed method achieves more accurate SAR image registration with lower RMSE and STD under different scenes.

Generally speaking, the computation time will become longer when the image size is larger or when there are many feature points. Table 1 shows that the increase in computation time of the proposed method is not overly long for images of agricultural land, fluvial area and Bali’s airport. However, it is longer for images of a suburban district and local area in Nantong because the image size is larger. The difference in computation time between the proposed method and KAZE-SAR is not significant. The time-consuming part of KAZE-SAR is the nonlinear diffusion filtering during scale-space construction, while for the proposed method, it is the edge point extraction and feature description.

In conclusion, the proposed method can register images with either few features, severe noise, different illuminations or different scales. The RMSE and STD of the proposed method are the lowest among the tested methods under different scenes.

## 4. Conclusions

A novel SAR image registration method, combing nonlinear diffusion filtering, Hessian features and edge points, is proposed in this paper. To preserve image edges and details while suppressing speckle noise, the proposed method employs the infinite symmetric exponential filter (ISEF) for image pre-processing and nonlinear diffusion filtering for scale-space construction. The ROEWA operator is used for image gradient computation to reduce the interference of multiplicative noise. In addition, Hessian features and edge points are adopted as image features to fully utilize the feature information.

The three sets of SAR image registration experiments demonstrate that, compared with SIFT-OCT, SAR-SIFT, Harris-SIFT, NF-Hessian and KAZE-SAR, the proposed method can provide reliable matching features and achieve more accurate registration results under different noise levels, geometric transformations and image scenes. Moreover, the proposed method has a broader range of applications than the aforementioned algorithms. It successfully attains the lowest RMSE and STD on SAR images with few corners, severe noise, different illuminations or different scales. The limitation of the proposed method is that the computation time is long when an image is too large or has too many features.

## Figures and Tables

**Table 1:** Results of SAR image registration obtained by different methods under different scenes.

Method	Agricultural Land (401 × 401)				Fluvial Area (397 × 390)				Suburban District (666 × 670)			
	CMN	RMSE	STD	TIME/s	CMN	RMSE	STD	TIME/s	CMN	RMSE	STD	TIME/s
SIFT-OCT	3	/	/	4.867	6	/	/	4.897	7	1.530928	1.201595	9.045
SAR-SIFT	82	0.906384	0.382543	2.918	8	1.428933	1.119463	2.468	46	1.255914	0.927354	6.246
NF-Hessian	131	0.779859	0.437463	6.507	29	0.879993	0.559184	5.437	22	1.368461	0.741137	7.416
Harris-SIFT	92	0.884048	0.415336	19.960	30	0.844939	0.342128	8.474	25	1.253105	0.617304	9.016
KAZE-SAR	173	0.851995	0.482133	8.997	51	0.892695	0.312734	7.154	21	1.374765	0.772329	28.931
Proposed method	211	0.720294	0.417708	10.114	41	0.657621	0.246255	5.937	75	1.167664	0.569378	27.280
**Method**	**Local Area in Nantong (** **800 × 798)**				**Bali’s Airport (** **512 × 512)**							
	**CMN**	**RMSE**	**STD**	**TIME/s**	**CMN**	**RMSE**	**STD**	**TIME/s**				
SIFT-OCT	6	/	/	9.716	22	0.726741	0.417401	7.966				
SAR-SIFT	106	1.292902	0.856279	22.488	10	1.386305	0.848438	5.238				
NF-Hessian	64	1.502358	1.047026	14.243	4	/	/	8.546				
Harris-SIFT	62	1.497583	0.878560	14.373	46	0.786863	0.371649	11.444				
KAZE-SAR	154	1.485735	1.099217	72.692	3	/	/	10.546				
Proposed method	125	1.092320	0.470561	75.064	98	0.641220	0.285698	16.771				

/ indicates a failure of registration.

## Data Availability

Data are contained within the article.
